# *SEPT9* and *SHOX2* DNA methylation status and its utility in the diagnosis of colonic adenomas and colorectal adenocarcinomas

**DOI:** 10.1186/s13148-016-0267-5

**Published:** 2016-09-20

**Authors:** Alexander Semaan, Anne van Ellen, Sebastian Meller, Dominik Bergheim, Vittorio Branchi, Philipp Lingohr, Diane Goltz, Jörg C. Kalff, Glen Kristiansen, Hanno Matthaei, Dimitrios Pantelis, Dimo Dietrich

**Affiliations:** 1 Department of General, Visceral, Thoracic and Vascular Surgery, University of Bonn, Sigmund-Freud-Straße 25, 53127 Bonn, Germany; 2 Institute of Pathology, University of Bonn, Sigmund-Freud-Straße 25, 53127 Bonn, Germany

**Keywords:** *SHOX2*, *SEPT9*, Adenomas, Colorectal cancer, Methylation, Amplification

## Abstract

**Background:**

Colorectal cancer (CRC) appear to arise from precursor lesions in a well-characterized adenoma-carcinoma sequence. Significant efforts have been invested to develop biomarkers that identify early adenocarcinomas and adenomas with high-grade dysplasia, since these are believed to harbor a particularly high risk for malignant transition and thus require resection. Promoter methylation of *SEPT9* and *SHOX2* has been suggested as a biomarker for various solid malignant tumors. Hence, the present study aimed to test their biomarker potential in CRC and precursor lesions.

**Results:**

Assessment of promoter methylation of *SEPT9* distinguished adenomas and CRC from controls as well as advanced from non-advanced adenomas (all *p* < 0.001). Correspondingly, *SHOX2* methylation levels in adenomas and colorectal carcinomas were significantly higher compared to those in normal control tissues (*p* < 0.001). Histologic transition from adenomas to CRC was paralleled by amplification of the *SEPT9* gene locus.

**Conclusions:**

*SEPT9*/*SHOX2* methylation assays may help to distinguish colorectal cancer and adenomas from normal and inflammatory colonic tissue, as well as advanced from non-advanced adenomas. Further studies need to validate these findings before introduction in clinical routine.

## Background

Colorectal cancer (CRC) is one of the most common and intensively studied cancer entities worldwide [1]. Although its molecular pattern is to a certain extent heterogeneous, more than 80 % of sporadic CRC appear to arise from precursor lesions [2]. The “adenoma-carcinoma sequence” [3] reflects this transition with a genetic characteristic, e.g., mutations in *TP53*, *KRAS*, and *APC*, [4] and histopathologic alterations [5, 6]. The evolving knowledge about precancerous lesions of CRC, the oftentimes slow progression towards malignant transformation, and a tremendously better prognosis for early detected and treated CRCs make this cancer entity particularly attractive for screening strategies [7]. Owing to multimodal therapy regimes, improved surgical techniques, and screening programs, incidence of CRC has steadily decreased over the last decades [8, 9]. Despite this significant success, population’s enrollment in recommended screening programs is difficult [10] and significant efforts have been invested in the development of non-invasive diagnostic tests. Nonetheless, colonoscopy remains the non-replaceable gold standard in every CRC screening program [11, 12].

Epigenetic changes to genomic tumor DNA are biostable and often cancer-specific alterations that are therefore issue of numerous ongoing research projects worldwide [13]. Our and other groups established differential methylation-specific qPCR assays of the stature homeobox 2 (*SHOX2*) and septin 9 (*SEPT9*) in various cancer entities with possible biomarker properties for early detection and response prediction strategies [14]. For example, our team previously used a combined assay to measure *SHOX2* and *SEPT9* promoter methylation for the discrimination between benign and malignant pleural effusions [15]. Promoter methylation of *SEPT9*, a gene encoding for a GTP-binding protein with various functions in formation and control of the cytoskeleton, proved to be present in >90 % of CRC specimens (mean methylation level 26 %, range 0–89 %) [16–18]. Furthermore, *SEPT9* DNA methylation in blood plasma was successfully validated in a large prospective trial including ~8000 asymptomatic subjects, undergoing routine colonoscopy (ClinicalTrials.gov Identifier: NCT00855348) [19]. Consequently, *SEPT9*-based diagnostic tests for colorectal cancer screening are available to patients in Europe and the USA as CE-marked In Vitro Diagnostics (CE-IVDs) and Laboratory Developed Tests (LDTs). Very recently, the American Food and Drug Administration (FDA) approved the commercial *SEPT9* methylation assay, “Epi proColon®,” as a blood-based in vitro diagnostic (IVD) test for screening of CRC. However, the *SEPT9* methylation pattern of adenomas has only been studied in a small amount of studies [20–24]. It is known today that certain types of adenomas harbor an increased risk for malignant transformation [25] and that adenomatous subtypes carry different epigenetic profiles [26]. Up-to-date detailed information about the *SEPT9* methylation status in different types of adenomatous polyps is still fragmentary.

*SHOX2* harbors two large CpG islands, located at the 3′ and the 5′ end of the gene, and is involved in limb formation and cardiac development [27, 28]. So far, the *SHOX2* DNA methylation status has mainly been evaluated for its value in the detection of lung cancer with reasonable success [29–34]. More than 90 % of histologically confirmed lung cancer patients showed a hypermethylation of this gene locus in comparison to normal tissue [35]. Additionally, the *SHOX2* methylation status showed promising results in other cancer types [15, 36]. In order to probe the apparently broad utility of *SHOX2* as a biomarker in CRC, this target was included in the present study.

## Results

### Stepwise increase of SEPT9 methylation from non-cancerous to cancerous tissue

**Fig. 1:**
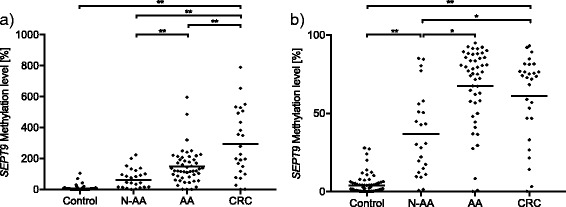
*SEPT9* methylation quantified with Triplex and QM assays, respectively, comparing the different subgroups (control vs. non-advanced vs. advanced adenomas vs. CRC). Comparison of different *SEPT9* methylation levels obtained from the **a** Triplex assay (control *n* = 62, N-AA *n* = 24, AA *n* = 48, CRC *n* = 25) and **b** QM assay (control *n* = 63, N-AA *n* = 24, AA *n* = 48, CRC *n* = 28). **p < 0.001 marks significant differences in methylation levels between the indicated subgroups, and *p < 0.05 marks significant differences in methylation levels between the indicated subgroups. *N-AA* non-advanced adenomas, *AA* advanced adenomas

In the Triplex assay, *SEPT9* promoter methylation levels showed a gradual increase from the control group (4.4 % ± 9.9), over non-advanced (N-AA, 72.7 % ± 63.3) and advanced adenomas (AA, 150.2 % ± 110.05), to CRC (294.8 % ± 219.2, Fig. 1a). The Triplex *SEPT9* methylation assay was able to differentiate between CRCs vs. controls (*p* < 0.001), CRCs vs. adenomas (*p* = 0.001) and CRC vs. AA (*p* < 0.001), CRC vs. N-AA (*p* < 0.001). Additionally, AA showed a hypermethylation compared to N-AA (*p* < 0.001) or control (*p* < 0.001).

**Fig. 2:**
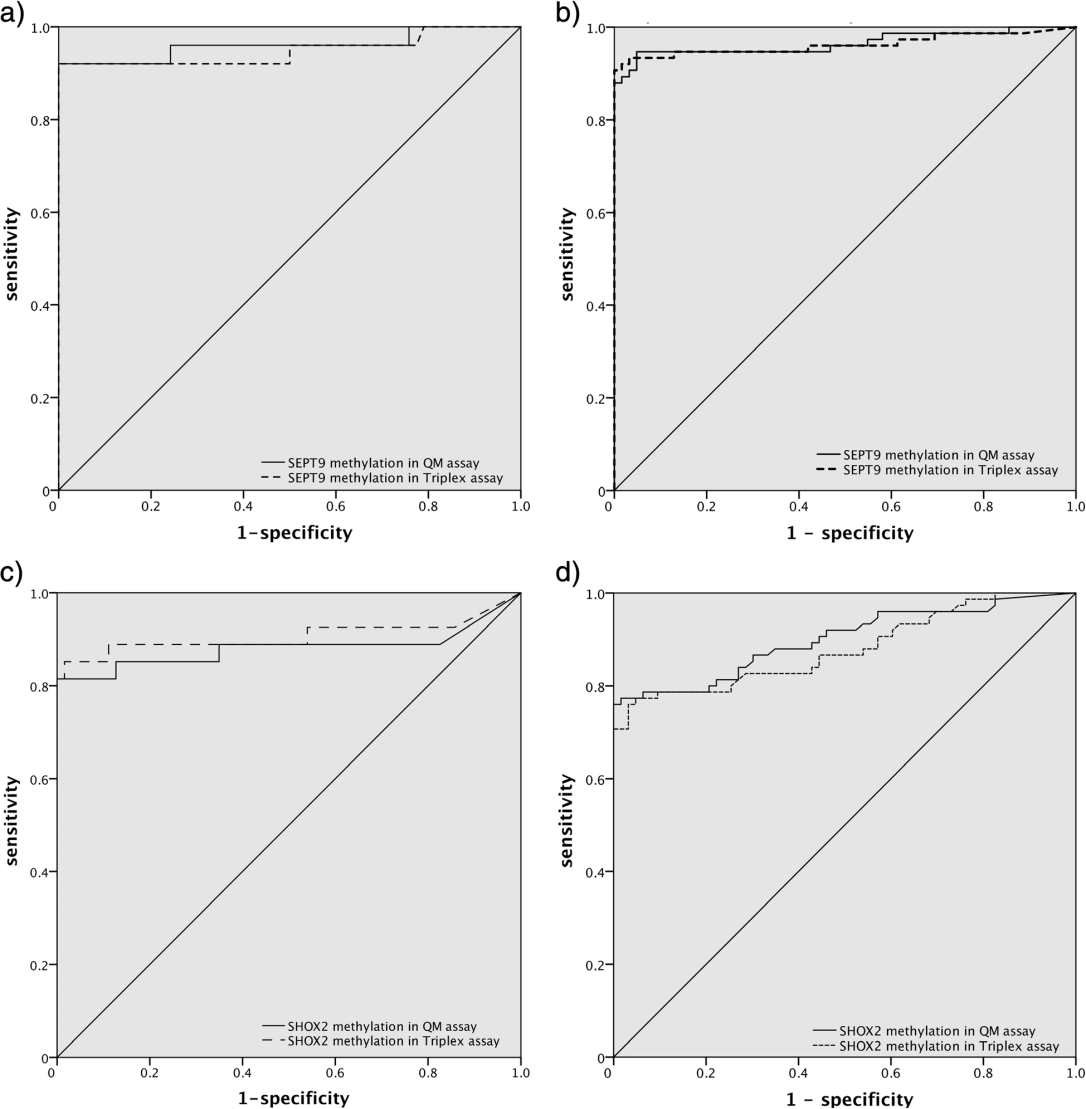
ROC curves for *SEPT9* and *SHOX2* methylation (determined via QM and Triplex assays) in the different subgroups. **a** *SEPT9* CRC vs. control. **b** *SEPT9* adenoma vs. control. **c** *SHOX2* CRC vs. control. **d** *SHOX2* adenoma vs. control

Receiver operating characteristic (ROC) analyses showed that *SEPT9* hypermethylation was able to distinguish between CRC vs. control (AUC = 0.96, 95 % CI = 0.90–1.00, *p* < 0.001) and adenomas vs. control (AUC = 0.97, 95 % CI = 0.93–1.00, both *p* < 0.001; Fig. 2a, b).

In total, 59/166 (35 %) specimens showed a *SEPT9* methylation level ≥100 % (highest methylation level of 789 %) and again a stepwise increment was noted from control to CRC. In the CRC group, 19/25 (76 %) showed a methylation level ≥100 %, whereas 39/75 (52 %) adenomas and 0/62 controls had a methylation level ≥100 %.

The QM assay also showed an increase in the *SEPT9* methylation level from the control group (2.4 % ± 3.2), over adenomas (55.8 % ± 29.3), to CRC (60.9 % ±26.9). N-AA (36.7 ± 26.6 and AA 67.5 ± 24.6) also showed an increase in the *SEPT9* methylation level (*p* < 0.001, Fig. 1b). Although the QM assay showed different methylation levels between CRC vs. controls (*p* < 0.001), CRC vs. N-AA (*p* = 0.002), and AA vs. controls (*p* < 0.001), this assay was not able to differentiate between CRC vs. AA (*p* = 0.29) and CRC vs. adenomas (*p* = 0.43).

ROC analyses showed that *SEPT9* hypermethylation was able to distinguish between CRC vs. control (AUC = 0.95, 95 % CI = 0.88–1.00, *p* < 0.001) and adenomas vs. control (QM: AUC = 0.96, 95 % CI = 0.93–0.99, *p* < 0.001; Fig. 2a, b).

### SHOX2 methylation shows a gradual increase from non-cancerous tissues to CRC

**Fig. 3:**
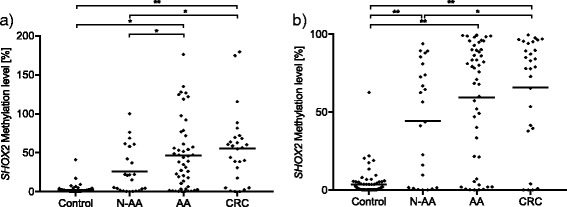
*SHOX2* methylation quantified with Triplex and QM assays, respectively, comparing the different subgroups (control vs. adenomas vs. CRC). Comparison of different *SHOX2* methylation levels obtained from the **a** Triplex assay (control *n* = 62, adenomas *n* = 75, CRC *n* = 25) and the **b** QM assay (control *n* = 63, adenomas *n* = 75, CRC *n* = 28) for the three subgroups. **p < 0.001 marks significant differences in methylation levels between the indicated subgroups, and *p < 0.05 marks significant differences in methylation levels between the indicated subgroups

As shown for the *SEPT9* gene locus, *SHOX2* showed a gradual step-up in the methylation level from control (1.3 % ± 1.5), over N-AA (26.3 % ± 29.6) and AA (46.7 % ± 44.9), to CRC (65.7 % ± 35.4), in the Triplex assay. Adenomas showed a *SHOX2* methylation level of 40.2 % ± 40.6 without separation in N-AA and AA. *SHOX2* methylation level was able to discriminate CRC vs. control (*p* < 0.001), CRC vs. N-AA (*p* = 0.01), N-AA vs. AA (*p* = 0.025), AA vs. control (*p* < 0.001), and N-AA vs. control (*p* < 0.001). Nonetheless, this assay was not able to distinguish between CRC vs. AA (*p* = 0.44) and CRC vs. adenoma (*p* = 0.12, Fig. 3).

ROC analyses showed that *SHOX2* hypermethylation was able to discriminate between CRC vs. controls (AUC = 0.91, 95 % CI = 0.81–1.00, *p* < 0.001) and adenomas vs. controls (AUC = 0.88, 95 % CI = 0.82–0.94, *p* < 0.001, Fig. 2c, d). However, *SHOX2* methylation assay failed in the differentiation between CRC and adenoma (AUC = 0.60, 95 % CI = 0.48–0.74, *p* = 0.095).

In total, 11/166 (7 %) specimens showed a *SHOX2* methylation level in the Triplex assay ≥100 % (highest methylation level of 179 %). There was no difference between the groups with 3/27 (11 %) in the CRC group, 8/75 (11 %) in the adenoma group, and 0/63 in the control group.

The QM assay also showed an increase of the *SHOX2* methylation level from controls (1.5 % ± 2.3), over N-AA (44.3 % ± 36.3) and AA (59.5 % ± 37.2), to CRC (65.7 % ± 35.4). Adenomas showed a *SHOX2* methylation level of 55.3 % ± 36.9 without separation in N-AA and AA. *SHOX2* methylation level was able to discriminate CRC vs. control (*p* < 0.001), CRC vs. N-AA (*p* = 0.036), AA vs. control (*p* < 0.001), and N-AA vs. control (*p* < 0.001). Nonetheless, this assay was not able to separate CRC from N-AA, AA, or adenomas (all *p* > 0.05) or AA from N-AA (*p* > 0.05, Fig. 3).

ROC analyses showed that *SHOX2* hypermethylation was able to discriminate between CRC vs. controls (AUC = 0.88, 95 % CI = 0.77–0.99, *p* < 0.001) and adenomas vs. controls (AUC = 0.90, 95 % CI = 0.85–0.95, *p* < 0.001, Fig. 2c, d). However, *SHOX2* methylation assay failed in the differentiation between CRC and adenoma (AUC = 0.56, 95 % CI = 0.43–0.69, *p* = 0.33).

### *SEPT9* and *SHOX2* methylation status in advanced adenomas

For three patients with colorectal adenoma, no information regarding size and grade of dysplasia could be retrieved, narrowing the amount of individuals in this analysis to 72.

In correlation to the stepwise increase of *SEPT9* and *SHOX2* methylation level, the adenoma group was evaluated for other characteristics with known potential for supporting a malignant transition.

In the Triplex assay, size ≥10 mm was significantly correlated with the methylation level of *SEPT9* (*ρ* = 0.40, *p* < 0.001), but showed no difference in the methylation level of *SHOX2* (*ρ* = 0.21, *p* = 0.083). Interestingly, those adenomas larger than ≥10 mm in size (*n* = 48) showed more frequently *SEPT9* methylation levels ≥100 % than adenomas ≤10 mm (*n* = 24) (33/48 (68 %) vs. 6/24 (25 %), *p* < 0.001). No difference between small and large adenomas was found regarding *SHOX2* methylation levels ≥100 % (*p* = 0.19). Correspondingly, the highest grade of epithelial dysplasia (D1-3) [37] is correlated with significantly higher methylation of *SEPT9* (*ρ* = 0.24, *p* = 0.047), but not of *SHOX2* (*ρ* = −0.06, *p* = 0.58).

In the QM methylation assay, size ≥10 mm was correlated with the methylation level of *SEPT9* (*ρ* = 0.48, *p* < 0.001) and *SHOX2* (*ρ* = 0.24, *p* = 0.047). The highest grade of epithelial dysplasia (D1-3) [37] showed a trend of higher methylation of *SEPT9* (*ρ* = 0.23, *p* = 0.056), but not of *SHOX2* (*ρ* = −0.06, *p* = 0.60). Adenomas with a size ≥10 mm had a higher *SEPT9* methylation level (67.5 vs. 36.7 %, *p* < 0.001) compared to small adenomas, but no difference was found in *SHOX2* (59.5 vs. 44.3 %, *p* = 0.10).

### *SEPT9* and *SHOX2* methylation levels in adenoma subtypes

**Fig. 4:**
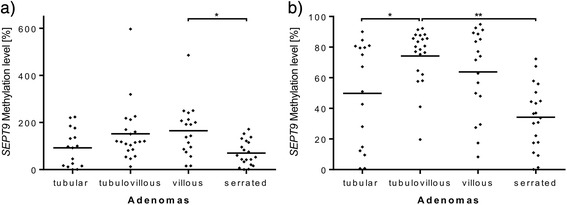
*SEPT9* methylation quantified with QM and Triplex assays, respectively, comparing different adenoma subtypes. Comparison of different *SEPT9* methylation levels in the **a** Triplex and **b** QM assays for the four adenomatous subgroups (tubular *n* = 16, tubulovillous *n* = 21, villous *n* = 18, serrated *n* = 20). **p < 0.001 marks significant differences in methylation levels between the indicated subgroups, and *p < 0.05 marks significant differences in methylation levels between the indicated subgroups

Regarding the methylation status of the *SEPT9* gene locus, (tubulo-)villous adenomas showed the highest methylation levels of all adenomas, irrespective of assay type. Consequently, villous adenomas showed a significant hypermethylation of *SEPT9* (Triplex assay) in comparison to serrated adenomas (164.9 vs. 70.3 %, *p* = 0.022, Fig. 4). All other types of adenomas showed no difference in methylation using the Triplex assay. According to results from the Triplex assay, QM assay analysis also showed a significant hypomethylation of serrated adenomas compared to villous adenomas (QM 34.2 vs. 63.7 %, *p* = 0.003; Triplex 70.3 vs. 164.9 %, *p* = 0.022) and tubulovillous adenomas (QM 34.2 vs. 74.2 %, *p* < 0.001; Triplex 70.3 vs. 152.0 %, *p* = 0.051). Additionally, tubulovillous in comparison to tubular adenomas showed a different *SEPT9* methylation level (QM 74.2 vs. 49.8 %, *p* = 0.029) (Fig. 4).

**Fig. 5:**
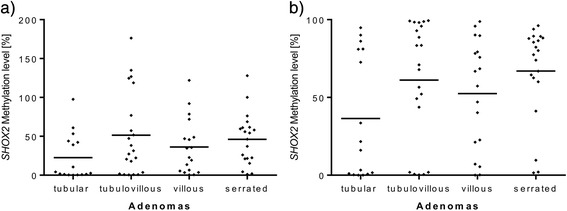
*SHOX2* methylation quantified with QM and Triplex assays, respectively, comparing different adenoma subtypes. Comparison of different *SHOX2* methylation levels obtained from the **a** Triplex and **b** QM assays for the four adenomatous subgroups (tubular *n* = 16, tubulovillous *n* = 21, villous *n* = 18, serrated *n* = 20). No significance level *p* < 0.05 has been reached in these assays

In contrast to *SEPT9*, methylation levels of the *SHOX2* gene locus revealed no difference between the various types of adenomas in both methylation assays (QM: villous vs. tubulovillous, villous vs. serrated, and villous vs. tubular, all *p* > 0.5; tubulovillous vs. tubular, *p* = 0.26 and serrated vs. tubular, *p* = 0.082) (Triplex: villous vs. tubulovillous, villous vs. serrated, villous vs. tubular, and serrated vs. tubular, all *p* > 0.5; tubulovillous vs. tubular, *p* = 0.20) (Fig. 5).

### Microsatellite instability

No correlation between any type of microsatellite instability (MSI) (MLH1, PMS2, MSH2, and MSH6), amplification of *SEPT9* and *SHOX2*, and clinical parameters were found (all *p* > 0.5).

## Discussion

Large screening programs have decreased the incidence of CRC [8, 9], but low compliance rates especially regarding preventive colonoscopy hamper a maximum success of modern primary prevention strategies [11]. The principle of “tailored screening” may improve screening effectiveness by stratification into risk tiers [38]. Therefore, entirely non-invasive early detection tests for CRC are urgently needed.

Epigenetic alternations of the *SEPT9* gene locus were previously described in minor series of tissue from colorectal adenomas [20–23] or blood samples [24, 39]. The presented comprehensive data confirmed previous findings and highlight the relevance of SEPT9 methylation in these lesions. Moreover, it was revealed that the assessment of promoter methylation of *SEPT9* as well as *SHOX2* may actually help distinguish CRC and adenomas from normal epithelium without dysplasia. These results are in line with Tänzer et al. who found a higher frequency of SEPT9 and ALX4 DNA methylations in plasma of AA compared to healthy controls [23]. On top of that, the methylation status of *SEPT9* as quantified with our Triplex assay was able to distinguish between adenomas vs. CRC and AA vs. N-AA. Despite their statistical significance, the methylation level of both genes within the subgroup showed a scattered and partly overlapping distribution, elevating the risk of false positive or negative results. These observations are in line with the finding that epigenetic changes resemble an early event in the carcinogenesis of CRC [40, 41]. In accordance, genome-wide comprehensive methylation analysis of adenomas and CRC tissue revealed accumulation of epigenetic alternations in the progression from early adenomas towards invasive adenocarcinomas [42, 43]. Although, advanced adenomas are believed to harbor an increased potential for malignant transformation (see [44] and [25]), a direct proof of malignant transformation of advanced adenomas or of a certain histologic subtype remains somewhat elusive.

Interestingly, a stepwise increase of the *SEPT9* methylation level from controls over not-advanced adenomas to advanced adenomas and invasive adenocarcinoma could be observed. This may explain false positive test results of the Epi proColon® test indicating methylated DNA segments in the *SEPT9* region in the absence of an invasive CRC. *SEPT9* may therefore potentially be used as an ancillary marker in the identification of advanced adenomas in case of difficulties with histologic diagnosis of a colorectal biopsy (i.e., due to cautery artifacts). Additionally, the presented data support a future inclusion of *SEPT9* and *SHOX2* in methylation biomarker panels to support the diagnosis of CRC, though both markers have to be validated in a large prospective trial. By combining these and other markers, overlapping results especially between AA and CRC may be minimized.

Surprisingly, >50 % of *SEPT9* Triplex methylation levels in CRC and adenomas showed a value exceeding 100 %, technically reasoned by a distant location of the reference gene (*ACTB* at chromosome 7) and the analyzed gene loci (*SEPT9* gene at chromosome 17 and *SHOX2* at chromosome 3q) [15]. Hence, higher methylation values in comparison to QM assay may be due to focal amplification of the *SEPT9* gene locus and/or a deletion of the reference gene (*ACTB*). A *SEPT9* amplification later during carcinogenesis is supported by the observation that especially advanced adenomas and carcinomas showed a level >100 % in the Triplex assay. These findings are in congruence with the results from Ben-David et al. who found that genes, which have been up-regulated in early adenomas, showed a tendency for amplification in later stages of colorectal carcinogenesis [45]. Taken together, one may carefully speculate that promoter hypermethylation of *SEPT9* triggers a focal amplification that may foster a malignant transition from adenoma to invasive carcinoma. This mirrors the assumption of Saha et al. and Bardelli et al. that gene amplification is attributed to play a role in stage transition of CRC [46, 47]. Concordantly, *SEPT9* amplification has only been identified in advanced breast cancer patients [48] and in vitro [49], while early localized tumors showed no *SEPT9* amplification. Additionally, the same group was able to identify a worse survival in endometrial adenocarcinoma, which shows a copy number variation (CNV) of *SEPT9* [50]. In contrast to this hypothesis, *SEPT9* amplification was only found in 0.4 % of CRC in a cohort of 257 CRC patients and in 2/615 CRC cases of the TCGA data repository (0.3 %) [4]. If the above assumptions are proven true, *SEPT9* may be an excellent plasmatic biomarker. Because, due to DNA amplification, the total number of tumor DNA alleles in plasma including their specific epigenetic *SEPT9* alteration may be easier to detect among background DNA. This phenomenon has already been evidenced for *SHOX2* in squamous cell lung cancer [34]. Another possible explanation for this phenomenon was hypothesized to be a MSI, which is known for causing hyper-mutated and epigenetically altered genomes [4]. However, no correlation between MSI and amplifications of *SEPT9* and *SHOX2* or clinical parameters were found in the present study.

Overall, *SEPT9* methylation showed a better performance than *SHOX2* in distinguishing the different study groups. This finding is in line with results about the potential of plasmatic free-circulating tumor DNA and tissue *SEPT9* methylation [17–19, 51–53], although sensitivity (52–94 %) and specificity (88–95 %) vary greatly between the studies [17, 18, 51, 54]. This variability may be attributed to different testing methods or common pre-existing conditions like high age, gender, and comorbidities and have to be interpreted with caution [53, 55].

The present study has several limitations. For example, only a small series was included and analyzed in a retrospective fashion hampering statistical power and lowering the evidence level. Furthermore, methylation assays have only a limited suitability to detect CNV, and more sensitive assays (e.g., FISH, CGH, or next-generation sequencing) have to validate the data.

## Conclusions

In conclusion, *SEPT9* or *SHOX2* methylation may be auxiliary biomarkers for the differentiation of CRC and advanced adenomas to non-advanced adenomas and normal tissue. Ideally, both markers should be integrated into a marker panel for CRC screening and validated in a large prospective trial. The investigations suggest that CNV of *SEPT9* may contribute to a malignant transition from adenomas into advanced adenomas and adenocarcinomas. Further studies with CNV sensible assays are needed to elucidate the distinct role of these gene loci in the carcinogenesis of CRC and their potential as biomarker.

## Methods

### Patient samples

The study was approved by the Institutional Review Board (IRB) of the University Hospital of Bonn (Number: 222/13). Formalin-fixed, paraffin-embedded (FFPE) tissue specimens from 166 patients, treated or diagnosed at the University Hospital of Bonn academic hospitals between 2002 and 2013, were included. The study group included various non-invasive epithelial lesions including tubular adenomas, tubulovillous adenomas, villous adenomas, and serrated adenomas. Furthermore, sporadic colorectal cancer were included. Patients with a history of familial adenomatous polyposis (FAP), Lynch syndrome, or other second primary malignancy were not included. Additionally, patients with Crohn’s disease showing high-grade dysplasia in the resected specimen or in any biopsy taken at time of admission were excluded from the study. All specimens were histologically diagnosed by an experienced pathologist blinded to the patient’s history. Histologic classification was performed according to the most recent recommendations by the World Health Organization [56] and the latest TNM classification [57]. Advanced adenomas were defined as adenomatous polyps with a size ≥10 mm, ≥25 % of villous features, or a high-grade dysplasia [58–61].

### Patient characteristics

The study cohort comprised tissue specimens from 166 patients (102 ♂, 64 ♀) with a mean age of 67 years (range 38–91). Age and gender were distributed equally in the following groups (all *p* > 0.5). The CRC group embodied 28 patients (18 ♂, 10 ♀) with a mean age of 69 years (range 42–90). This group included 7 stage I, 7 stage IIa, 3 stage IIb, 3 stage IIIa, 1 stage IIIb, 4 stage IIIc, and 3 stage IV CRCs. The adenoma group contained 75 individuals (45 ♂, 30 ♀) with a mean age of 68 years (range 38–89). Sixteen of the adenomas were described as tubular, 21 as tubulovillous, 18 as villous, and 20 as serrated adenomas. Twenty-four of 75 adenomas (32 %) measured <1 cm, and 48/75 (64 %) had a size >1 cm, while 3 datasets were missing (4 %). The mean size of adenomas was 2 cm, the median 1.5 cm (range 0.3–8 cm). Fifty-three adenomas of 75 (70.7 %) showed a low or moderate grade of dysplasia, 19/75 (25.3 %) showed high-grade dysplasia, while 3 datasets were missing (4 %). The control group comprised of 63 patients (39 ♂, 24 ♀) with a mean age of 66 years (range 39–91). This group contained 34 normal adjunct tissue specimens (NAT), 23 specimens diagnosed with Crohn’s disease and admitted for surgical colon resection because of obstruction, abscesses, or fistula and 6 specimens with colonic diverticulosis.

Valid Triplex assay measurements were obtained from 25 CRCs; 75 adenomas, including 16 tubular, 21 tubulovillous, 18 villous, and 20 serrated adenomas; and 62 controls.

Valid QM assay measurements were obtained from 28 CRC, 75 adenoma (16 tubular, 21 tubulovillous, 18 villous, and 20 serrated adenomas), and 63 control specimens.

### TCGA data

Data plots for CNV were conducted using cBioPortal (http://www.cbioportal.org/index.do) [62, 63] and the gene-centric GISTIC analyses provided at http://www.broadinstitute.org/tcga/home [64]. These results are in whole based upon data generated by the TCGA Research Network (http://cancergenome.nih.gov/).

### DNA extraction and methylation analysis

DNA extraction was performed using the Bisulfite All-In-One Kit (innuCONVERT, Analytik Jena, Germany). The detailed protocol is described elsewhere [65, 66]. Locus-specific analyses of DNA methylation patterns were performed using two different PCR methods of methylation analyses for each specimen: (1) Triplex methylation-specific qPCR (Triplex qMSP) [15] and (2) quantitative methylation PCR (QM PCR) [67].

#### SHOX2/SEPT9/ACTB Triplex assay

As previously described, a methylation-specific Triplex qPCR assay was used [15]. It quantifies the number of methylated *SHOX2* and *SEPT9* alleles, referred to total DNA copy numbers. The total DNA copy number was quantified using a qPCR assay targeting the β-actin (*ACTB*) gene locus comprising no CpG sites [15]. A calibrator sample (bisulfite-converted artificially methylated DNA) was used in order to allow for an accurate quantification as previously described [15].

#### QM assay

A modified quantitative methylation real-time PCR, called QM PCR assay [67], was used. It allows a simultaneous amplification of methylated and unmethylated alleles in a single tube. The composition of the PCR buffer has been described earlier [34]. Primers (*SHOX2*-forward: cctcctaccttctaaccc, *SHOX2*-reverse: gttttttggatagttaggtaat, *SEPT9*-forward: aaataatcccatccaacta, *SEPT9*-reverse: gttgtttattagttattatgt) and probes (*SHOX2*-methylated: 6-FAM-ctcgtacgaccccgatcg-BBQ650, *SHOX2*-unmethylated: HEX-tactcatacaaccccaatcaaaca-BHQ1, *SEPT9*-methylated: 6-FAM-ttaaccgcgaaatccgac-BHQ1, *SEPT9*-unmethylated: HEX-acattaaccacaaaatccaac-BHQ1) were applied in a final concentration of 0.6 μM each. PCR was performed with an AB 7500 Fast Real-Time PCR System (Life Technologies Corporation, Carlsbad, CA, USA) using the following temperature profile: 15 min at 95 °C of initial denaturation followed by 45 cycles with 15 s, 95 °C and 60 s, 60 °C. As a calibrator sample for the QM PCR assay, a 50 % mixture of bisulfite-converted artificially methylated DNA (CpGenome™ Universal Methylated DNA; Merck Millipore, Darmstadt, Germany) and unmethylated DNA (NW Andrology & Cryobank Inc., Spokane, WA, USA) was used.

#### Calculation of methylation levels

The calibrator and all the samples were analyzed in triplicate (Triplex assay) and in duplicate (QM assay), respectively. The mean average of the CT values was calculated and used for further analysis. Previously described adapted ∆∆CT methods were applied to determine a relative methylation value from the QM assay [68] and the Triplex assay [15] measurements. Invalid PCR results indicated by high cycle threshold (CT) values (CT_*ACTB*_ > 33 (Triplex assay); CT_methylated allele_ > 33 and CT_unmethylated allele_ > 33 (QM assay)) were omitted from the analysis.

### Immunohistochemical staining for MSI

Tumors with MSI are known for their hyper-mutated genome and epigenetic alternations [4]. To analyze *SHOX2/SEPT9* in association with MSI, immunohistochemical staining (IHC) was used to estimate the protein expression levels of the four major mismatch repair enzymes (*MLH1*, *PMS2*, *MSH2*, and *MSH6*). For this purpose, tissue microarrays (TMA) were assembled from formalin-fixed, paraffin-embedded tissue. For each case (adenoma, carcinoma, control group), representative areas were marked on the hematoxylin-eosin-stained section. Subsequently, at least 1-mm cores were punched and arrayed in a paraffin block. IHC stainings were performed with a Ventana Benchmark-automated staining system (Ventana Medical Systems, Tucson, AZ, USA), following the manufactures’ protocol. The following antibodies from BD Pharmingen were used: *MLH-1* #550838 clon G168-15 (1:50), *MSH-2* #556349 mouse anti-human, *MSH6* BD Pharmingen #610918 mouse anti-human, and *PMS2* BD Pharmingen #556415 mouse monoclonal. The slides were then counterstained with hematoxylin, dehydrated, and mounted. Presence of mismatch repair enzymes was then evaluated by an experienced pathologist.

### Availability of data and materials

The TCGA dataset supporting the conclusions of this article is available in the TCGA data repository (http://cancergenome.nih.gov/). The dataset supporting the conclusions of this article is included within the article and its figures.

### Data evaluation and statistical analysis

Values are expressed as mean or median, unless otherwise stated. Differences between groups were tested using *t* test, Mann-Whitney *U* test, or ANOVA, where appropriate. Correlation between values was tested using Spearman’s rank correlation. The Bonferroni correction was used in case of multiple pairwise comparisons. Receiver operating characteristic (ROC) curves were calculated to observe the ability of the methylation level of *SHOX2* and *SEPT9* gene loci to differentiate between the subgroups. Areas under the curve (AUCs) were reported. *p* values of <0.05 were considered to be statistically significant. Statistical analyses were performed with SPSS (IBM, Armonk, NY, USA, version 23) and GraphPad Prism 4 (GraphPad Software Inc., San Diego, CA, USA).
